# Cholesterol Metabolism and Weight Reduction in Subjects with Mild Obstructive Sleep Apnoea: A Randomised, Controlled Study

**DOI:** 10.1155/2013/769457

**Published:** 2013-05-16

**Authors:** Maarit Hallikainen, Henri Tuomilehto, Tarja Martikainen, Esko Vanninen, Juha Seppä, Jouko Kokkarinen, Jukka Randell, Helena Gylling

**Affiliations:** ^1^Institute of Public Health and Clinical Nutrition, Department of Clinical Nutrition, University of Eastern Finland, P.O. BOX 1627, 70211 Kuopio, Finland; ^2^Institute of Clinical Medicine, Department of Otorhinolaryngology, Kuopio University Hospital, and University of Eastern Finland, P.O. BOX 1777, 70211 Kuopio, Finland; ^3^Oivauni Sleep Clinic, Puijonkatu 12 b, 70100 Kuopio, Finland; ^4^Institute of Clinical Medicine, Department of Medicine, Division of Clinical Nutrition, Kuopio University Hospital, P.O. BOX 1777, 70211 Kuopio, Finland; ^5^Institute of Clinical Medicine, Clinical Physiology and Nuclear Medicine, Kuopio University Hospital, and University of Eastern Finland, P.O. BOX 1777, 70211 Kuopio, Finland; ^6^Institute of Clinical Medicine, Respiratory Medicine, Kuopio University Hospital, and University of Eastern Finland, P.O. BOX 1777, 70211 Kuopio, Finland; ^7^Division of Internal Medicine, Department of Medicine, University of Helsinki, Helsinki, P.O. BOX 700, 00029 HUS, Finland

## Abstract

To evaluate whether parameters of obstructive sleep apnoea (OSA) associate with cholesterol metabolism before and after weight reduction, 42 middle-aged overweight subjects with mild OSA were randomised to intensive lifestyle intervention (*N* = 23) or to control group (*N* = 18) with routine lifestyle counselling only. Cholesterol metabolism was evaluated with serum noncholesterol sterol ratios to cholesterol, surrogate markers of cholesterol absorption (cholestanol and plant sterols) and synthesis (cholestenol, desmosterol, and lathosterol) at baseline and after 1-year intervention. At baseline, arterial oxygen saturation (Sa_O_2__) was associated with serum campesterol (*P* < 0.05) and inversely with desmosterol ratios (*P* < 0.001) independently of gender, BMI, and homeostasis model assessment index of insulin resistance (HOMA-IR). Apnoea-hypopnoea index (AHI) was not associated with cholesterol metabolism. Weight reduction significantly increased Sa_O_2__and serum cholestanol and decreased AHI and serum cholestenol ratios. In the groups combined, the changes in AHI were inversely associated with changes of cholestanol and positively with cholestenol ratios independent of gender and the changes of BMI and HOMA-IR (*P* < 0.05). In conclusion, mild OSA seemed to be associated with cholesterol metabolism independent of BMI and HOMA-IR. Weight reduction increased the markers of cholesterol absorption and decreased those of cholesterol synthesis in the overweight subjects with mild OSA.

## 1. Introduction

Obstructive sleep apnoea (OSA) characterized by repeated episodes of apnoea and hypopnoea during sleep is one of the most common sleep disturbances [1]. OSA is independently associated with hypertension, cardiovascular diseases, metabolic syndrome, insulin resistance, and type 2 diabetes [2–7]. Furthermore, recent epidemiological studies have concluded that OSA is an important risk factor for mortality, particularly due to coronary artery disease [8, 9]. However, the underlying mechanisms explaining these associations are rather complex, and although several possibilities have been proposed, they are not entirely accepted. In general, atherogenesis as well as OSA is considered as slow processes, and the onset is likely to begin years before any symptoms appear. We have earlier demonstrated that even mild OSA is associated with the activation of the proinflammatory system [10]. Furthermore, since elevated LDL cholesterol level is one of the most important risk factors for cardiovascular diseases, the question raises whether OSA has a role in hypercholesterolaemia or in cholesterol metabolism. In some, but not in all studies, OSA has independently associated with increased concentrations of total cholesterol and triglycerides and decreased concentrations of HDL cholesterol [11–14]. The mechanisms of dyslipidaemia in OSA besides obesity are not clearly understood especially for elevated LDL cholesterol level [15], but most likely chronic intermittent hypoxemia (IH), a major component of OSA, may be the primary trigger for a cascade of pathogenetic mechanisms leading to increased triglyceride-rich lipoproteins and reduced HDL cholesterol levels [15]. Regarding cholesterol metabolism there are no clinical studies examining the association between OSA and cholesterol metabolism (i.e., cholesterol synthesis and absorption). 

The most important risk factor for OSA is obesity [16]. On the other side, obesity interferes with cholesterol metabolism, so that cholesterol synthesis is upregulated, and cholesterol absorption efficiency is low [17, 18]. Accordingly, it could be assumed that cholesterol metabolism might be disturbed in OSA, but whether it is obesity or OSA that interferes with cholesterol metabolism remains to be evaluated. However, this does not change the fact that 60–90% of all patients with OSA are obese [19] and need to be treated not only for OSA but also other obesity related comorbidities. 

It was recently demonstrated that lifestyle intervention with weight reduction reduced both hypopnoea and especially apnoea indices and also other obesity related risk factors for cardiovascular diseases in a vast majority of patients with mild OSA, highlighting the importance of an early lifestyle intervention [20]. Similarly, weight reduction decreases cholesterol synthesis and increases cholesterol absorption in type 2 diabetics [21, 22]. It would be interesting to know whether in subjects with OSA weight reduction alters also cholesterol metabolism, and whether the reduction in apnoea and hypopnoea indices are related to cholesterol metabolism beyond obesity. Therefore, in the present randomised interventional study two main parameters of OSA, that is, apnoea-hypopnoea index (AHI) and arterial oxygen saturation (Sa_O_2__), were related to those of cholesterol synthesis and absorption at baseline and after one-year weight reduction program in middle-aged overweight subjects with mild OSA. Cholesterol metabolism was evaluated with serum noncholesterol sterols, surrogate markers of cholesterol absorption and synthesis [23].

## 2. Methods

This study is a substudy of our randomised study originally conducted to determine the effects of changes in lifestyle with weight reduction program designed to prevent the progression of the disease or even cure it in the most prevalent subgroup of OSA, that is, overweight patients with mild OSA. The detailed design of the study was previously reported [20]. 

### 2.1. Subjects

The subjects were consecutively recruited from among patients referred to the outpatient clinics of Otorhinolaryngology and Respiratory Medicine of Kuopio University Hospital, Finland, because of a suspicion of OSA. The main study population consisted of 72 subjects who completed the 1-year follow-up [20]. The inclusion criteria were age 18–65 years, overweight (BMI ≥ 28 kg/m^2^), and mild OSA (AHI 5–15 events/h). The exclusion criteria were active treatment of OSA of any kind, pregnancy, chronic kidney, thyroid, or liver disease. To the present study, an additional inclusion criterion was the availability of both baseline and follow-up measurements, and an additional exclusion criterion was the presence of lipid-lowering medication. 

This study was conducted according to the guidelines laid down in the Declaration of Helsinki, and all procedures involving human subjects were approved by the Research Ethics Committee of the Hospital District of Northern Savo (Kuopio, Finland). All subjects gave their written informed consent for the study. 

### 2.2. Intervention

A detailed description of the intervention procedure was previously reported by Tuomilehto et al. [20]. The subjects with mild OSA were randomised to two groups. The subjects in the intervention group were provided with a group-based very low calorie diet (VLCD) of 600–800 kcal/d for 12 weeks, after which they were advised regarding diet and exercise. The lifestyle intervention lasted for 1 year and consisted of 14 visits with the study nutritionist. The subjects in the control group were given standard care consisting of general oral and written information about diet and exercise at baseline and 3-month visits by the study nurse and physician without any specific individualised advice. 

### 2.3. Measurements

Nocturnal cardiorespiratory monitoring by Embletta (Embla, Broomfield, CO, USA) at home was conducted in accordance with accepted guidelines for diagnosing OSA [24]. Apnoea was defined as a cessation (>90%) of airflow for >10 s with oxygen desaturation for ≥4%. Hypopnoea was defined as a reduction (>30%) of airflow for >10 s with oxygen desaturation for ≥4%. AHI was defined as the number of apnoeas and hypopnoeas per hour, and mild OSA was defined as an AHI of 5–15 events/h [24]. Furthermore, mean arterial oxygen saturation (Sa_O_2__) and time and percentage with arterial oxygen saturation <90% were assessed. The recordings were manually evaluated by two blinded, trained physicians. 

Body weight was measured with a digital scale and height using a stadiometer. A trained nurse measured also waist circumference both at the baseline and at the 1-year visit.

Blood samples for biochemical assays were collected from fasting subjects (≥12 h). Serum total and HDL cholesterol, serum triglycerides and plasma glucose were analysed by using automated analyzer system (Konelab 60 Analyzer, ThermoFisher Scientific, Waltham, MA, USA). LDL cholesterol was calculated according to Friedewald equation. Plasma glucose was analysed by using automated analyzer system (Konelab 60 Analyzer, ThermoFisher Scientific, Waltham, MA, USA). Serum insulin was measured by using a fluoroimmunoassay system (Wallac, Perkin-Elmer, Waltham, MA, USA). The homeostasis model assessment index of insulin resistance (HOMA-IR) was calculated as fasting serum insulin concentration × fasting plasma glucose concentration/22.5 [25].

Serum cholesterol, cholesterol precursors reflecting cholesterol synthesis (squalene, cholestenol, lathosterol, and desmosterol), plant sterols (sitosterol and campesterol), and cholestanol reflecting cholesterol absorption [23] were quantified from nonsaponifiable serum material by GLC (Agilent 7890GC System, Agilent Technologies, Wilmington, DE, USA) equipped with a 50 m long Ultra 1 capillary column (5% Phenyl-methyl siloxane) (Agilent Technologies, Wilmington, DE, USA) [26]. Serum values were expressed in terms of 10^2^x mmol/mol of cholesterol (called ratio in the text) by dividing the noncholesterol sterol concentrations with the cholesterol value of the same GLC run to eliminate the changing concentrations of lipoproteins (mainly LDL) that transports noncholesterol sterols. Ratios of relative synthesis markers/absorption markers were also calculated reflecting cholesterol metabolism.

### 2.4. Statistical Analyses

Statistical analyses were performed with SPSS for Windows 14.0 statistics program (SPSS, Chicago, IL, USA).

 Normality and homogeneity of variance assumptions were checked before further analyses. Student's *t*-test was used to compare the baseline values and the changes between the groups. ANOVA for repeated measurements was used to analyse the interaction of time and group and changes over time in between-group comparisons followed by post hoc comparisons with Bonferroni corrections. In between-group comparisons, gender and BMI, were included as ANCOVA. For some variables of interest Pearson or Spearman correlation coefficients were calculated. In addition, to evaluate the effects of gender, BMI and HOMA-IR on associations of parameters of OSA and cholesterol metabolism, a multiple linear regression analysis was used. Variables not normally distributed even after logarithmic transformation, nonhomogenous in variance, or noncontinuous were tested with Mann-Whitney *U* test or Fisher exact test. A *P* value of <0.05 was considered statistically significant. The results are given as means ± SEM. 

## 3. Results

### 3.1. Baseline

A total of 41 subjects (33 men and 8 women) fulfilled the criteria and were included into the statistical analyses. Their mean age was 48.9 ± 1.3 years and BMI 32.5 ± 0.4 kg/m^2^. Thirteen subjects had antihypertensive medication, two had thyroxin therapy, and one had oral diabetes medication. Baseline characteristics of the subjects in the control (*N* = 18) and intervention (*N* = 23) groups are shown in Table 1. Despite randomisation, the subjects in the intervention group were heavier, and their BMI was higher and waist circumference larger than in the control group. In addition, serum insulin concentration and cholestenol : cholesterol ratio were greater, and HOMA-IR tended to be significantly greater (*P* = 0.055) compared with controls (Table 2). No other significant differences between the groups were observed.

#### 3.1.1. Baseline Associations

In the whole population, cholesterol synthesis markers were interrelated (e.g., cholestenol versus lathosterol *r* = 0.572, *P* < 0.001) as well as the absorption markers (campesterol versus cholestanol *r* = 0.664, *P* < 0.001). Cholesterol synthesis markers were inversely associated with the absorption markers (e.g., desmosterol versus cholestanol *r* = −0.637, *P* < 0.001) suggesting that cholesterol homeostasis was intact. 

The markers of cholesterol synthesis were positively (e.g., desmosterol ratio *r* = 0.364, *P* = 0.020) and those of cholesterol absorption inversely (e.g., cholestanol ratio *r* = −0.376, *P* = 0.020) associated with BMI. The cholesterol synthesis markers were inversely associated with serum HDL cholesterol concentration (e.g., desmosterol ratio *r* = −0.502, *P* = 0.001) and positively with serum triglycerides (e.g., desmosterol ratio *r* = 0.546, *P* < 0.001), whereas the associations between the absorption markers and HDL cholesterol (e.g., cholestanol ratio *r* = 0.316, *P* = 0.040) and triglycerides (e.g., cholestanol ratio *r* = −0.343, *P* = 0.028) were opposite. No associations of the cholesterol synthesis and absorption markers with total and LDL cholesterol were found. 

Mean total AHI was not associated with mean arterial oxygen saturation (Sa_O_2__) or with any other variables, even though AHI tended to be associated with serum cholestenol ratio to cholesterol (*r* = 0.292, *P* = 0.064). 

Sa_O_2__ was inversely associated with body weight (*r* = −0.656, *P* < 0.001) and waist circumference (*r* = −0.481, *P* = 0.003) and positively with HDL cholesterol concentration (*r* = 0.410, *P* = 0.010). Sa_O_2__ was inversely associated with desmosterol (*r* = −0.595, *P* < 0.001) and positively with campesterol ratios to cholesterol (*r* = 0.381, *P* = 0.020). In multiple linear regression analysis after adjustment with gender, BMI and HOMA-IR, the associations between Sa_O_2__ and desmosterol and campesterol ratios to cholesterol and desmosterol : campesterol ratio remained significant (Table 3).

### 3.2. Intervention

#### 3.2.1. Anthropometric Measurements

BMI and waist circumference reduced significantly more in the intervention group compared with the control group (−13.6% versus −2.2% and −10.9% versus −2.2%, resp.) (Table 1) during the follow-up. 

#### 3.2.2. Serum Cholesterol, Plasma Glucose, and Serum Insulin

Plasma glucose was similarly reduced in both study groups (*P* < 0.05), but the reduction was not significant after adjustment with gender and BMI (Table 1). The reduction in serum insulin concentration was greater in the intervention group compared with the controls (−43.6% versus −17.8%, *P* < 0.05), but the intervention values did not differ between the groups (Table 1). Similarly, the percentage reduction in HOMA-IR was greater in the intervention group compared with the controls (−46.7% versus −19.5%, *P* < 0.05).

#### 3.2.3. Serum Lipids

Serum total and LDL cholesterol concentrations were not changed during the intervention (Table 1). Serum HDL cholesterol concentration significantly increased in the intervention group, and the percentage increase was greater than in the control group. However, the intervention values did not differ between the groups (Table 1). Serum triglyceride concentration was similarly reduced in both study groups (*P* < 0.05), but the reductions were not significant after adjustment with gender and BMI (Table 1). 

#### 3.2.4. Cardiorespiratory Recordings

The mean total AHI at 12 months and the mean percentage change (−49.1% versus +30.9%) during the follow-up significantly differed between the groups (Table 1). However, after adjustment with gender and BMI the mean total AHI only tended to be lower in the intervention group compared with controls (*P* = 0.052). A significant improvement was detected in mean Sa_O_2__ in the intervention group compared with the control group at 12 months (Table 1). In addition, the subjects in the intervention group spent less time with Sa_O_2__ < 90% during their sleep compared with subjects in the control group (Table 1). 

#### 3.2.5. Cholesterol Synthesis and Absorption Markers

Of the cholesterol synthesis markers, serum cholestenol ratio to cholesterol was significantly reduced by −18.1% in the intervention group, and the change was significantly different compared with the controls (Table 2). Serum desmosterol and lathosterol ratios to cholesterol similarly reduced in both groups (*P* < 0.05), but the reductions were not significant after adjustment with gender and BMI (Table 2). No changes in serum squalene were found (Table 2).

Of the cholesterol absorption markers, serum cholestanol : cholesterol ratio significantly increased by 11.4% during the follow-up in the intervention group, and the change was significantly different compared with the controls (Table 2). However, the intervention values did not differ between the groups (Table 2). Serum campesterol and sitosterol ratios to cholesterol did not change (Table 2). 

The changes in the concentrations of serum cholesterol synthesis and absorption markers were in line with the respective changes in the ratio to cholesterol (data not shown). 

Serum cholestenol, desmosterol, and lathosterol ratios to cholestanol significantly reduced by −19.6–−25.4% during the follow-up in the intervention group, and the changes significantly differed from controls, but only the intervention values of cholestenol : cholestanol ratio significantly differed compared with controls (Table 2).

#### 3.2.6. Associations during Intervention (Intervention + Control Groups Combined)

**Figure 1:**
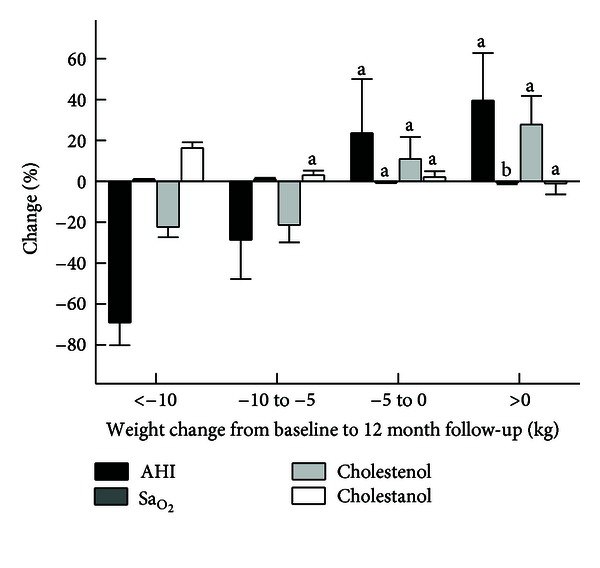
The percentage changes in apnoea-hypopnoea index (AHI), mean oxygen saturation (Sa_O_2__), and cholestenol and cholestanol : cholesterol ratios (10^2^x mmol/mol of cholesterol) in relation to changes in body weight: less than −10 kg, −10 to −5 kg, and −5 to 0 kg and more than 0 kg ^a^ *P* < 0.05 or less from weight reduction of <−10 kg, ^b^ *P* < 0.05 or less from weight reduction of −5 to 0 kg.

Figure 1 shows the percentage changes in AHI, Sa_O_2__, cholestenol, and cholestanol : cholesterol ratios in relation to weight reduction. The greater the weight reduction, the greater the reductions in AHI and serum cholestenol : cholesterol ratio and the increases in Sa_O_2__ and serum cholestanol : cholesterol ratio.

**Figure 2:**
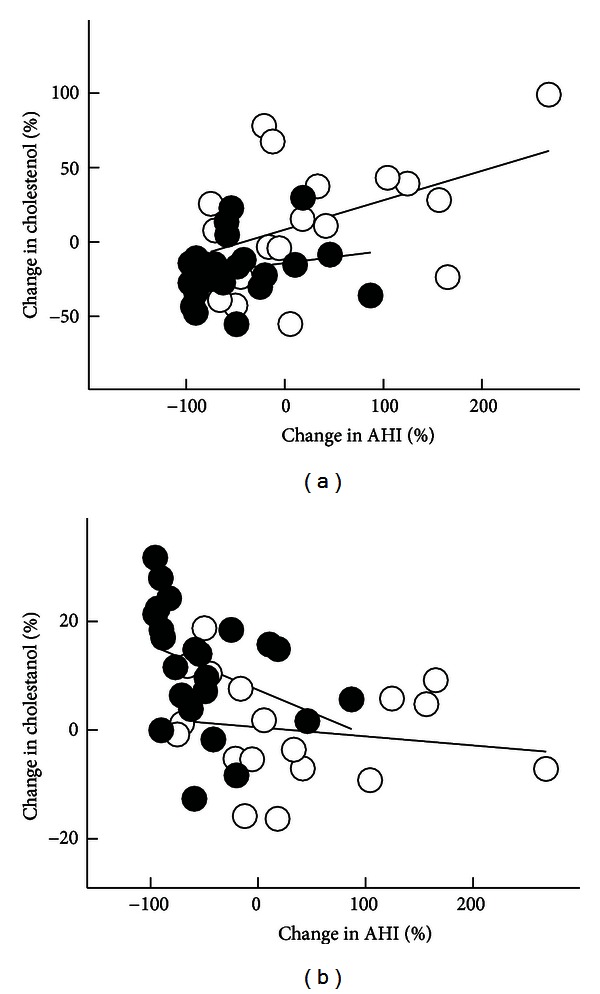
The associations between the percentage changes in apnoea-hypopnoea index (AHI) and serum cholestenol ((a), *r* = 0.589, *P* < 0.001, *N* = 41) and cholestanol ratios to cholesterol (10^2^ x mmol/mol of cholesterol) ((b), *r* = −0.441, *P* = 0.004, *N* = 41) in the control (○) and intervention (●) groups.

The percentage change of AHI was positively associated with the respective changes of serum synthesis markers (e.g., serum cholestenol : cholesterol ratio, Figure 2(a)) and inversely with the percentage change of serum cholestanol : cholesterol ratio (Figure 2(b)). After adjustment with gender and the percentage changes of BMI and HOMA-IR, the associations between the percentage changes of AHI and cholestanol : cholesterol ratio (*P* = 0.004) and of the cholesterol synthesis markers cholestenol : cholesterol (*P* = 0.021) remained significant. The positive associations between the percentage changes of AHI and cholestenol, desmosterol, and lathosterol ratios to cholestanol (*r* = 0.460–0.519, *P* = 0.001–0.003) remained significant after adjustment with gender and the percentage changes of BMI and HOMA-IR, too. However, the inverse associations between the percentage changes of Sa_O_2__ and cholestenol and desmosterol ratios to campesterol (*r* = −0.365–−0.375, *P* = 0.024–0.026) did not remain significant after adjustment with gender and the percentage changes of BMI and HOMA-IR. 

## 4. Discussion

The novel observations of the present study were that mild OSA seems to be associated with cholesterol metabolism independent of BMI and HOMA-IR. Secondly, one-year weight reduction program resulted in changes in cholesterol metabolism. Thirdly, after weight reduction the improvement in OSA was associated with changes in cholesterol metabolism independent of weight and HOMA-IR reductions. 

Weight reduction achieved by intensive lifestyle counselling with an initial VLCD program increased Sa_O_2__ and serum cholestanol : cholesterol ratio suggesting that cholesterol absorption was increased. In addition, AHI and serum cholestenol : cholesterol ratio was decreased suggesting that cholesterol synthesis was downregulated. Similar findings regarding cholesterol metabolism were reported in in type 2 diabetics [21, 22]. The present study also demonstrated that all these changes sustained up to 1 year. The negative interrelations of cholesterol synthesis and absorption markers at baseline and after one year (data not shown) suggest that cholesterol homeostasis remained intact irrespective of the intervention. 

There is a definite need for further research to better understand the links between OSA and lipid metabolism and to improve the current guidelines of treatment. Thus far, the interventional studies examining the effects of OSA treatment on lipid metabolism have exclusively been conducted with continuous positive airway pressure (CPAP). However, the evidence of the effects of CPAP treatment on dyslipidaemia is limited. In one recent study, CPAP has been demonstrated to reduce postprandial serum triglyceride and total cholesterol concentrations [27] and in another, fasting serum cholesterol, but not triglyceride concentrations [28]. The consensus statement of International Diabetes Federation highly recommends further research on OSA and metabolism in general and particularly intervention studies with emphasis on cardiovascular risk factors, also without using CPAP [29]. The statement concludes that management of OSA should focus initially on weight reduction for the overweight and obese. In general, weight reduction improves serum lipid profile [30]. In the present study, weight reduction did not change serum total and LDL cholesterol concentrations even though it affected cholesterol metabolism. 

In animal models, IH was suggested to induce dyslipidaemia by upregulating biosynthesis of VLDL in liver, increasing lipolysis in adipocytes, and inhibiting lipoprotein clearance [31–34]. Recently, in patients with OSA, hepatic expression of stearoyl CoA desaturase, a key enzyme of lipid biosynthesis, has been demonstrated to increase in direct proportion to the severity of nocturnal IH [33]. In addition, overexpression was associated with marked increase in plasma triglyceride and LDL cholesterol concentrations [33]. However, thus far there is no consistent evidence supporting the relationship between OSA and dyslipidaemia in humans [11–14].

Based on animal models, IH was suggested not to affect cholesterol biosynthesis, because there was no effect on the key genes involved in cholesterol biosynthesis [32]. However, we found in the subjects with mild OSA that Sa_O_2__, one of the key cardiorespiratory variables, was positively associated with cholesterol absorption (serum campesterol : cholesterol ratio) and inversely with cholesterol synthesis (serum desmosterol : cholesterol ratio) at baseline suggesting that the greater Sa_O_2__, the greater the cholesterol absorption and the lower the cholesterol synthesis. These associations were independent from gender, BMI and HOMA-IR. In addition, although AHI was not significantly associated with cholesterol absorption markers, it tended to be associated positively with cholesterol synthesis markers (cholestenol : cholesterol ratio, *P* = 0.062). 

In the whole study population, the greater the weight reduction, the greater the reductions in AHI and serum cholestenol : cholesterol ratio and the increases in   Sa_O_2__ and serum cholestanol : cholesterol ratio. In addition, the percentage change of AHI was inversely associated with that of cholesterol absorption marker (cholestanol) and positively with the percentage change of cholesterol synthesis markers. This suggests that the more AHI was improved, the more cholesterol absorption was increased and cholesterol synthesis decreased. The associations between cholestanol and of the cholesterol synthesis marker cholestenol and AHI remained significant after adjustment with gender and the percentage changes of BMI and HOMA-IR. On the contrary, the relationships between the percentage changes of Sa_O_2__ and cholesterol absorption and synthesis markers were to opposite directions than with AHI, but the relationships did not reached statistical significance.

Even mild OSA is associated with increased activation of the inflammatory system and a risk for cardiovascular morbidity, although the risk is more frequently associated with more severe degrees of the disease [8–10]. The exact underlying mechanisms explaining the association between cardiovascular morbidity and OSA are not fully understood, although a multifactorial aetiology is most likely [6]. It is suggested that OSA may accelerate atherosclerosis affecting the key risk factors of atherosclerosis. One of the proposed potential mediators and accelerators of atherosclerosis in OSA is postulated to be dysregulation of lipid metabolism and dyslipidaemia. Cholesterol absorption is low, and cholesterol synthesis is high in obesity [17], insulin resistance [35], and type 2 diabetes even without obesity [36]. In type 2 diabetes, weight reduction increased cholesterol absorption and downregulated cholesterol synthesis simultaneously with improved glucose balance [22]. Therefore, low absorption-high synthesis of cholesterol might be proatherogenic in insulin-resistant situations, possibly frequently present also in OSA. This kind of metabolic profile of cholesterol of insulin resistance is different from that observed in primary hypercholesterolaemia, in which high absorption-low synthesis of cholesterol is associated with unfavorable prognosis during statin treatment in coronary subjects [37]. The present study demonstrates that with weight reduction both OSA and the possibly proatherogenic profile of cholesterol metabolism can be improved. The intriguing question, however, is by which mechanism the variables of OSA and cholesterol metabolism are interrelated beyond obesity. Both at baseline and after the intervention with improved tissue oxygenation the associations between OSA and cholesterol metabolism were similar: better oxygenation is associated with higher absorption and lower synthesis of cholesterol independent of BMI. 

Our study population was homogenous consisting only of overweight subjects with mild OSA. Therefore, the results cannot be generalized to all OSA patients, and there exist also some limitations in this study. The sample size per group was relatively small, and it was smaller than in the original study [20], because the subjects using lipid-lowering medication had to be excluded. With larger study population the results, which now tended to be significant, might become significant. Furthermore, the sample size of the present study is similar to those in other recent interventional studies examining lipid metabolisms in OSA patients. Because no stratification in terms of BMI was used in the randomisation, the weight between the groups differed at baseline. Therefore, we adjusted the results for the baseline BMI. Furthermore, because serum insulin concentration and cholestenol : cholesterol ratio were greater and HOMA-IR tended to significantly greater in the intervention group compared with controls at baseline, the baseline values were taken into account as covariance in the analyses of those variables. Regardless of some limitations, the present study is, as far as we know, the first study to examine the associations between OSA and cholesterol metabolism in humans and with and without lifestyle intervention in a randomised settings. Furthermore, this study demonstrated the even mild OSA seemed to be associated with abnormal cholesterol metabolism.

CPAP is considered as a “gold standard” of OSA treatment [38]. However, the adherence of some patients to CPAP treatment is unsatisfactory, particularly in the early stages of the OSA. Based on the recent randomised studies and on the fact that obesity is the most important risk factor for OSA and most OSA patients are obese, weight reduction by lifestyle changes (healthy eating habits, food behaviour therapy if needed and physical activity) should be the first line or at least part of the treatment for all overweight OSA patients. Besides improving OSA, weight reduction seems to improve also other obesity-related disturbances of cardiometabolic syndrome [20, 39].

In conclusion, weight reduction with intensive lifestyle counselling with an initial VLCD program increased the markers of cholesterol absorption and decreased those of cholesterol synthesis in subjects with mild OSA. High Sa_O_2__ seems to be associated with high cholesterol absorption and low Sa_O_2__ with upregulated cholesterol synthesis independent of BMI, HOMA-IR, and gender. Improvement in AHI was associated with increased cholesterol absorption and decreased cholesterol synthesis independent of BMI and HOMA-IR. However, further studies with greater number of subjects are needed to confirm these results.

## Figures and Tables

**Table 1:** Changes in anthropometric measurements, plasma glucose and serum insulin concentrations, and cardiorespiratory variables during the intervention.

	Control group		Intervention group		*P* ^a^	*P* ^b^
	Baseline	12 months	Baseline	12 months		
Gender (M/F)	14/4		19/4		0.500	
Age (y)	48.2 ± 2		49.4 ± 1.7		0.667	
Weight (kg)	93.9 ± 2.9	92.2 ± 3.4	103.5 ± 2.2^c^	91.5 ± 2.4^d,e^	<0.001	<0.001
BMI (kg/m^2^)	31.4 ± 0.6	30.9 ± 0.9	33.4 ± 0.6^c^	29.5 ± 0.6^d,e^	<0.001	<0.001
Waist circumference (cm)	104.6 ± 2.3	102.9 ± 2.6	112.9 ± 2.1^c^	101.0 ± 2.0^d,e^	<0.001	<0.001
P-Glucose (mmol/L)^(f)^	6.19 ± 0.51	5.57 ± 0.16	5.92 ± 0.19	5.59 ± 0.13	0.528	0.306
S-Insulin (mU/L)	10.18 ± 1.06	8.25 ± 1.05	14.61 ± 1.69^c^	7.16 ± 0.63	0.082	0.105
HOMA-IR	2.66 ± 0.33	2.08 ± 0.28	3.98 ± 0.54	1.80 ± 0.18	0.087	0.103
Total cholesterol (mmol/L)	5.17 ± 0.25	5.21 ± 0.23	4.83 ± 0.11	4.63 ± 0.16	0.246	0.369
LDL cholesterol (mmol/L)	3.13 ± 0.17	3.28 ± 0.17	3.06 ± 0.10	2.88 ± 0.14	0.116	0.226
HDL cholesterol (mmol/L)	1.13 ± 0.10	1.12 ± 0.07	0.98 ± 0.05	1.15 ± 0.06^d^	0.028	0.044
Total triglycerides (mmol/L)^(f)^	1.78 ± 0.24	1.60 ± 0.30	1.73 ± 0.17	1.32 ± 0.12	0.683	0.510
AHI (total)	9.5 ± 0.8	11.2 ± 2.0	9.8 ± 0.6	5.2 ± 1.2^d,(e)^	0.007	0.040
Mean Sa_O_2__	94.5 ± 0.3	93.9 ± 0.3^d^	94.0 ± 0.3	94.8 ± 0.3^d,e^	<0.001	0.001
Time with Sa_O_2__ < 90%	3.5 ± 0.9	9.2 ± 3.5	6.4 ± 2.2	4.7 ± 2.1^d,e^	0.007	0.004
Percentage time with Sa_O_2__ < 90%	0.9 ± 0.2	2.3 ± 0.9^d^	1.6 ± 0.5	0.8 ± 0.3^d,e^	0.003	0.002

Values shown are means ± SEM.

^a^Group by time interaction analysed with analysis of variance for repeated measurements (GLM). If the baseline differed between groups (*P* < 0.06), it was taken into account as covariance. (Gender Fisher's exact test and age Student's *t*-test).

^b^Group by time interaction (gender and BMI as covariance).

^c^ *P* < 0.05 denotes a significant difference at the baseline between the groups.

^d^ *P* < 0.05 denotes a significant difference from the baseline within the groups.

^e^ *P* < 0.05 denotes a significant difference at the 12 months between the groups, ^(e)^ *P* > 0.05 (gender and BMI as covariance).

^f^ *P* < 0.05 denotes a significant change over time, ^(f)^ *P* > 0.05 (gender and BMI as covariance).

**Table 2:** Changes in serum noncholesterol sterols and squalene during the intervention.

	Control group		Intervention group		*P* ^a^	*P* ^b^
	Baseline	12 months	Baseline	12 months		
Cholesterol (mg/dL)	195.9 ± 9.6	193.9 ± 9.3	181.9 ± 5.2	172.5 ± 5.6	0.219	0.413
Cholesterol synthesis markers						
Squalene : cholesterol	26.5 ± 2.6	24.7 ± 2.7	23.5 ± 1.2	22.1 ± 1.7	0.860	0.408
Cholestenol : cholesterol	15.2 ± 1.4	16.9 ± 1.9	22.3 ± 2.9^c^	18.0 ± 2.9^d,e^	0.025	0.020
Desmosterol : cholesterol^(f)^	66.3 ± 4.5	63.4 ± 5.2	77.8 ± 5.0	65.6 ± 2.8	0.131	0.161
Lathosterol : cholesterol^(f)^	179.4 ± 14	175.1 ± 12.7	208.3 ± 13.0	178.9 ± 14.1	0.126	0.069
Cholesterol absorption markers						
Campesterol : cholesterol	206.1 ± 27.7	208.2 ± 18.3	227.1 ± 24.2	235.1 ± 30.6	0.656	0.670
Sitosterol : cholesterol	108.1 ± 11.6	107.5 ± 7.7	132.5 ± 16.0	136.6 ± 16.1	0.843	0.842
Cholestanol : cholesterol	131.1 ± 5.8	130.6 ± 6.1	121.0 ± 4.6	135.5 ± 6.7^d^	0.002	0.004
Cholesterol metabolism markers						
Squalene : Cholestanol	0.21 ± 0.03	0.20 ± 0.03	0.20 ± 0.01	0.18 ± 0.02	0.293	0.109
Cholestenol : Cholestanol	0.12 ± 0.01	0.14 ± 0.02	0.20 ± 0.03^c^	0.14 ± 0.02^d,e^	0.006	0.006
Desmosterol : Cholestanol	0.54 ± 0.06	0.52 ± 0.06	0.68 ± 0.06	0.52 ± 0.04^d^	0.016	0.029
Lathosterol : Cholestanol	1.42 ± 0.12	1.42 ± 0.13	1.81 ± 0.15	1.45 ± 0.17^d^	0.026	0.017

Values shown are means ± SEM.

^a^Group by time interaction analysed with analysis of variance for repeated measurements (GLM). If the baseline differed between groups (*P* < 0.05), it was taken into account as covariance.

^b^Group by time interaction (gender and BMI as covariance).

^c^ *P* < 0.05 denotes a significant difference at the baseline between the groups.

^d^ *P* < 0.05 denotes a significant difference from the baseline within the groups.

^e^ *P* < 0.05 denotes a significant difference at the 12 months between the groups.

^f^ *P* < 0.05 denotes a significant change over time ^(f)^ *P* > 0.05 (gender and BMI as covariance).

**Table 3:** Associations between serum desmosterol and campesterol ratios to cholesterol and desmosterol : campesterol ratio and Sa_O_2__*.

	Desmosterol : cholesterol		Campesterol : cholesterol		Desmosterol : campesterol	
	Beta	*P* value	Beta	*P* value	Beta	*P* value
Gender	−0.264	0.062	−0.348	0.094	0.131	0.742
BMI (kg/m^2^)	0.047	0.732	−0.033	0.873	0.045	0.802
HOMA-IR	0.524	<0.001	−0.011	0.956	0.247	0.153
Sa_O_2__	−0.322	0.031	0.604	0.008	−0.584	0.004
*R* ^2^	0.623		0.175		0.360	

*Multiple linear regression analysis models (adjustment with gender, BMI, and HOMA-IR).

## References

[1] Young T, Peppard PE, Gottlieb DJ (2002). Epidemiology of obstructive sleep apnea: a population health perspective. *American Journal of Respiratory and Critical Care Medicine*.

[2] Coughlin SR, Mawdsley L, Mugarza JA, Calverley PMA, Wilding JPH (2004). Obstructive sleep apnoea is independently associated with an increased prevalence of metabolic syndrome. *European Heart Journal*.

[3] Nieto FJ, Young TB, Lind BK (2000). Association of sleep-disordered breathing sleep apnea, and hypertension in a large community-based study. *Journal of the American Medical Association*.

[4] Peppard PE, Young T, Palta M, Skatrud J (2000). Prospective study of the association between sleep-disordered breathing and hypertension. *The New England Journal of Medicine*.

[5] Shahar E, Whitney CW, Redline S (2001). Sleep-disordered breathing and cardiovascular disease: cross-sectional results of the sleep heart health study. *American Journal of Respiratory and Critical Care Medicine*.

[6] Shamsuzzaman ASM, Gersh BJ, Somers VK (2003). Obstructive sleep apnea: implications for cardiac and vascular disease. *Journal of the American Medical Association*.

[7] Tuomilehto H, Peltonen M, Partinen M (2008). Sleep-disordered breathing is related to an increased risk for type 2 diabetes in middle-aged men, but not in women -the FIN-D2D survey. *Diabetes, Obesity and Metabolism*.

[8] Punjabi NM, Caffo BS, Goodwin JL (2009). Sleep-disordered breathing and mortality: A Prospective Cohort Study. *PLoS Medicine*.

[9] Partinen M, Guilleminault C (1990). Daytime sleepiness and vascular morbidity at seven-year follow-up in obstructive sleep apnea patients. *Chest*.

[10] Sahlman J, Miettinen K, Peuhkurinen K (2010). The activation of the inflammatory cytokines in overweight patients with mild obstructive sleep apnoea: sleep apnea and inflammation. *Journal of Sleep Research*.

[11] Newman AB, Nieto FJ, Guidry U (2001). Relation of sleep-disordered breathing to cardiovascular disease risk factors: The Sleep Heart Health Study. *American Journal of Epidemiology*.

[12] Roche F, Sforza E, Pichot V (2009). Obstructive sleep apnoea/hypopnea influences high-density lipoprotein cholesterol in the elderly. *Sleep Medicine*.

[13] Tsioufis C, Thomopoulos K, Dimitriadis K (2007). The incremental effect of obstructive sleep apnoea syndrome on arterial stiffness in newly diagnosed essential hypertensive subjects. *Journal of Hypertension*.

[14] Drager LF, Bortolotto LA, Lorenzi MC, Figueiredo AC, Krieger EM, Lorenzi-Filho G (2005). Early signs of atherosclerosis in obstructive sleep apnea. *American Journal of Respiratory and Critical Care Medicine*.

[15] Drager LF, Polotsky VY, Lorenzi-Filho G (2011). Obstructive sleep apnea: an emerging risk factor for atherosclerosis. *Chest*.

[16] Young T, Skatrud J, Peppard PE (2004). Risk factors for obstructive sleep apnea in adults. *Journal of the American Medical Association*.

[17] Miettinen TA, Gylling H (2000). Cholesterol absorption efficiency and sterol metabolism in obesity. *Atherosclerosis*.

[18] Gylling H, Hallikainen M, Kolehmainen M (2007). Cholesterol synthesis prevails over absorption in metabolic syndrome. *Translational Research*.

[19] Pillar G, Shehadeh N (2008). Abdominal fat and sleep apnea: the chicken or the egg?. *Diabetes Care*.

[20] Tuomilehto HPI, Seppä JM, Partinen MM (2009). Lifestyle intervention with weight reduction: first-line treatment in mild obstructive sleep apnea. *American Journal of Respiratory and Critical Care Medicine*.

[21] Simonen P, Gylling H, Miettinen TA (2002). Acute effects of weight reduction on cholesterol metabolism in obese type 2 diabetes. *Clinica Chimica Acta*.

[22] Simonen P, Gylling H, Howard AN, Miettinen TA (2000). Introducing a new component of the metabolic syndrome: low cholesterol absorption. *American Journal of Clinical Nutrition*.

[23] Miettinen TA, Tilvis RS, Kesäniemi YA (1990). Serum plant sterols and cholesterol precursors reflect cholesterol absorption and synthesis in volunteers of a randomly selected male population. *American Journal of Epidemiology*.

[24] Flemons WW, Buysse D, Redline S (1999). Sleep-related breathing disorders in adults: recommendations for syndrome definition and measurement techniques in clinical research. *Sleep*.

[25] Matthews DR, Hosker JP, Rudenski AS (1985). Homeostasis model assessment: Insulin resistance and *β*-cell function from fasting plasma glucose and insulin concentrations in man. *Diabetologia*.

[26] Miettinen TA (1988). Cholesterol metabolism during ketoconazole treatment in man. *Journal of Lipid Research*.

[27] Phillips CL, Yee BJ, Marshall NS, Liu PY, Sullivan DR, Grunstein RR (2011). Continuous positive airway pressure reduces postprandial lipidemia in obstructive sleep apnea: a randomized, placebo-controlled crossover trial. *American Journal of Respitatory and Critical Care Medicine*.

[28] Robinson GV, Pepperell JCT, Segal HC, Davies RJO, Stradling JR (2004). Circulating cardiovascular risk factors in obstructive sleep apnoea: data from randomised controlled trials. *Thorax*.

[29] Shaw JE, Punjabi NM, Wilding JP, Alberti KGMM, Zimmet PZ (2008). Sleep-disordered breathing and type 2 diabetes. A report from the International Diabetes Federation Taskforce on Epidemiology and Prevention. *Diabetes Research and Clinical Practice*.

[30] Dattilo AM, Kris-Etherton PM (1992). Effects of weight reduction on blood lipids and lipoproteins: a meta- analysis. *American Journal of Clinical Nutrition*.

[31] Li J, Savransky V, Nanayakkara A, Smith PL, O’Donnell CP, Polotsky VY (2007). Hyperlipidemia and lipid peroxidation are dependent on the severity of chronic intermittent hypoxia. *Journal of Applied Physiology*.

[32] Li J, Thorne LN, Punjabi NM (2005). Intermittent hypoxia induces hyperlipidemia in lean mice. *Circulation Research*.

[33] Savransky V, Jun J, Li J (2008). Dyslipidemia and atherosclerosis induced by chronic intermittent hypoxia are attenuated by deficiency of stearoyl coenzyme a desaturase. *Circulation Research*.

[34] Drager LF, Li J, Shin MK (2011). Intermittent hypoxia inhibits clearance of triglyceride-rich lipoproteins and inactivates adipose lipoprotein lipase in a mouse model of sleep apnoea. *European Heart Journal*.

[35] Pihlajamäki J, Gylling H, Miettinen TA, Laakso M (2004). Insulin resistance is associated with increased cholesterol synthesis and decreased cholesterol absorption in normoglycemic men. *Journal of Lipid Research*.

[36] Simonen PP, Gylling HK, Miettinen TA (2002). Diabetes contributes to cholesterol metabolism regardless of obesity. *Diabetes Care*.

[37] Miettinen TA, Gylling H, Strandberg T, Sarna S (1998). Baseline serum cholestanol as predictor of recurrent coronary events in subgroup of Scandinavian simvastatin survival study. *British Medical Journal*.

[38] Ballester E, Badia JR, Hernández L (1999). Evidence of the effectiveness of continuous positive airway pressure in the treatment of sleep apnea/hypopnea syndrome. *American Journal of Respiratory and Critical Care Medicine*.

[39] Johansson K, Hemmingsson E, Harlid R (2011). Longer term effects of very low energy diet on obstructive sleep apnoea in cohort derived from randomised controlled trial: Prospective Observational Follow-up Study. *British Medical Journal*.

